# Supplementary material for: Structure of PINK1 and mechanisms of Parkinson's disease-associated mutations
Source: eLife. 2017 Oct 5;6:e29985. doi: 10.7554/eLife.29985 (PMC5679756; doi:10.7554/eLife.29985)
Supplement: Supplementary file 1. [file elife-29985-supp1.docx]

| **Human**  **Mutation** | **Tribolium**  **Residue** | **Inheritance** | **Reference** | **Predicted Structural Impact** |
| --- | --- | --- | --- | --- |
| ­V170G | V176 | Homozygous | Moro (2008) Neurology 70, 1186 |  |
| A217D | A194 | Homozygous | Leutenegger (2006) Arch Neurol 63, 1257 | ATP-binding |
| E240K | E217 | Compound Heterozygous | Rogaeva (2004) Arch Neurol 61, 1898 | ATP-binding |
| A244G | A221 | Compound Heterozygous | Gelmetti (2008) Mov Disord 23, 881 | ATP-binding |
| H271Q | H247 | Homozygous | Hatano (2004) Ann Neurol 56, 424 |  |
| G309D | G285 | Homozygous | Valente (2004) Science 304, 1158 | Substrate-binding |
| L347P | L322 | Homozygous | Hatano (2004) Ann Neurol 56, 424 | Protein Stability |
| N367S | N342 | Compound Heterozygous | Choi (2008) Neurogenetics 9, 263 |  |
| I368N | L343 | Homozygous | Koziorowski (2013) Neurol Neurochir Pol 47, 319 | Protein Stability |
| L369P | L344 | Compound Heterozygous | Ibanez (2006) Brain 129, 686 | ATP-binding |
| G386A | G361 | Homozygous | Ibanez (2006) Brain 129, 686 | Catalytic/Activation loop |
| C388R | C363 | Homozygous | Li (2005) Neurology 64, 1955 | Protein Stability |
| G409V | G384 | Homozygous | Ibanez (2006) Brain 129, 686 | Protein Stability |
| P416R/L | P391 | Homozygous | Myhre (2008) BMC Neurol 8, 47  Lohmann (2012) Eur J Neurol 19, 769 | Catalytic/Activation loop |
| E417G | E392 | Homozygous | Hatano (2004) Ann Neurol 56, 424 | Catalytic/Activation loop |
| W437R/G/X | W412 | Homozygous | Kumazawa (2008) Arch Neurol 65, 802  Wilkins (2012) Parkinsonism Relat Disord 18, 386  Valente (2004) Science 304, 1158 | Protein Stability |
| G440E | G415 | Homozygous | Ishihara-Paul (2008) Neurology 71, 896 | Protein Stability |
| L489P | L462 | Compound Heterozygous | Rogaeva (2004) Arch Neurol 61, 1898 | Protein Stability |
| R492X | R465 | Compound Heterozygous | Hatano (2004) Ann Neurol 56, 424 | Protein Stability |
| L539F | L515 | Homozygous | Rafiqua Ben El Haj, 2016 | Protein Stability |
