# Supplementary material for: Structure of PINK1 and mechanisms of Parkinson's disease-associated mutations
Source: eLife. 2017 Oct 5;6:e29985. doi: 10.7554/eLife.29985 (PMC5679756; doi:10.7554/eLife.29985)
Supplement: Supplementary file 2. [file elife-29985-supp2.docx]

# Materials and Methods

**Primary Antibodies**

| **Antibody** | **Cat./Ref. No.** | **Company** | **Host** |
| --- | --- | --- | --- |
| FLAG (HRP) | A8592 | Sigma | Mouse |
| GAPDH (HRP) | 3683 | CST | Rabbit |
| pSer65 Ubiquitin | 3322 | In house | Rabbit |
| pSer65 Parkin | MJF-17 | ABCAM | Rabbit |
| Parkin | 32282 | SCB | Mouse |
| OPA-1 | 612607 | BD Biosciences | Mouse |
| Tribolium PINK1 | S121D (2^nd^ Bleed) | MRC-PPU Reagents | Sheep |

**Secondary Antibodies**

| **Antibody** | **Cat./Ref. No.** | **Company** | **Host** |
| --- | --- | --- | --- |
| anti-Mouse HRP conjugate | 31450 | Thermo Scientific | Rabbit |
| anti-Rabbit HRP conjugate | 31460 | Thermo Scientific | Goat |
| anti-Sheep HRP conjugate | 31480 | Thermo Scientific | Rabbit |
| Anti-Rabbit IRDye® 800CW conjugate | 926-32211 | LI-COR | Goat |

**cDNA clones used in present study**

| **Constructs** | **Reference DU no for mrcppu reagents** |
| --- | --- |
| pET15 6HIS SUMO *Tc*PINK1 (S150-D570, ΔI261-L270, S205E, E527A, K528A) | 51904 |
| MBP-*Tc*PINK1 wildtype | 34701 |
| MBP-*Tc*PINK1 D359A | 34832 |
| MBP-*Tc*PINK1 A194D | 57592 |
| MBP-*Tc*PINK1 K196A | 57593 |
| MBP-*Tc*PINK1 E217K | 38003 |
| MBP-*Tc*PINK1 delta261-270 | 51955 |
| MBP-*Tc*PINK1 S205A | 56425 |
| MBP-*Tc*PINK1 S207A | 56380 |
| MBP-*Tc*PINK1 S205A / S207A | 56356 |
| MBP-*Tc*PINK1 D381A | 57637 |
| MBP-*Tc*PINK1 R216A | 56540 |
| MBP-*Tc*PINK1 K212A | 56417 |
| MBP-*Tc*PINK1 R240A | 56435 |
| MBP-*Tc*PINK1 delta222-242 | 56478 |
| MBP-*Tc*PINK1 delta231-242 | 56479 |
| MBP-*Tc*PINK1 delta243-253 | 27662 |
| MBP-*Tc*PINK1 L371A | 57597 |
| MBP-*Tc*PINK1 Y375A | 57632 |
| MBP-*Tc*PINK1 K382A | 57599 |
| MBP-*Tc*PINK1 K435A | 27365 |
| 6His-SUMO-Parkin (res1-76) | 39607 |
| 6His-SUMO-Ubiquitin | 20027 |
| 6His-SUMO-*Tc*PINK1 (res125-end) D359A | 26039 |
| **Constructs used in HeLa cell experiments** | **DU** |
| 3FLAG empty vector | 45919 |
| hPINK1-3FLAG wildtype | 43407 |
| hPINK1-3FLAG D384A | 46669 |
| hPINK1-3FLAG A217D | 56028 |
| hPINK1-3FLAG K219A | 27432 |
| hPINK1-3FLAG E240K | 56079 |
| hPINK1-3FLAG L369P | 56042 |
| hPINK1-3FLAG delta180-209 | 27594 |
| hPINK1-3FLAG delta245-265 | 56564 |
| hPINK1-3FLAG delta285-294 | 56481 |
| Parkin | 23307 |
